# Wide variation in tissue, systemic, and drain fluid exposure after oxaliplatin-based HIPEC: results of the GUTOX study

**DOI:** 10.1007/s00280-020-04107-y

**Published:** 2020-06-27

**Authors:** Loek A. W. de Jong, Fortuné M. K. Elekonawo, Marie Lambert, Jan Marie de Gooyer, Henk M. W. Verheul, David M. Burger, Johannes H. W. de Wilt, Etienne Chatelut, Rob ter Heine, Philip R. de Reuver, Andre J. A. Bremers, Nielka P. van Erp

**Affiliations:** 1 Department of Pharmacy, Radboud Institute for Health Sciences, Radboud University Medical Center (RUMC), P.O. Box 9101, 6500 HB Nijmegen, The Netherlands; 2 Department of Radiology and Nuclear Medicine, Radboud Institute for Health Sciences, Radboud University Medical Center (RUMC), P.O. Box 9101, 6500 HB Nijmegen, The Netherlands; 3 Department of Surgery, Radboud Institute for Health Sciences, Radboud University Medical Center (RUMC), P.O. Box 9101, 6500 HB Nijmegen, The Netherlands; 4 Institut Claudius‑Regaud, IUCT‑Oncopole, and CRCT, Université de Toulouse, Inserm, 1, avenue Irène Joliot‑Curie, Toulouse, France; 5 Department of Medical Oncology, Radboud Institute for Health Sciences, Radboud University Medical Center (RUMC), P.O. Box 9101, 6500 HB Nijmegen, The Netherlands

**Keywords:** HIPEC, Hyperthermic intraperitoneal chemotherapy, Oxaliplatin, Peritoneal carcinomatosis, Colorectal peritoneal metastasis, Pharmacokinetics

## Abstract

**Purpose:**

In this exploratory study, the effect of postprocedural flushing with crystalloids after oxaliplatin-based hyperthermic intraperitoneal chemotherapy (HIPEC) on platinum concentrations in peritoneal tissue, blood, and drain fluid was studied. Interpatient variability in oxaliplatin pharmacokinetics and the relation between platinum concentration in peritoneal fluid and platinum exposure in tissue and blood was explored.

**Methods:**

Ten patients with peritoneal carcinomatosis of colorectal origin were treated with HIPEC including postprocedural flushing, followed by ten patients without flushing afterwards. Tissue, peritoneal fluid, blood, and drain fluid samples were collected for measurement of total and ultrafiltered platinum concentrations.

**Results:**

Peritoneal tissue concentration and systemic ultrafiltered platinum exposure showed large inter individual variability, ranging from 65 to 1640 µg/g dry weight and 10.5 to 28.0 µg*h/ml, respectively. No effect of flushing was found on geometric mean platinum concentration in peritoneal tissue (348 vs. 356 µg/g dry weight), blood (14.8 vs. 18.1 µg*h/ml), or drain fluid (day 1: 7.6 vs. 7.7 µg/ml; day 2: 1.7 vs. 1.9 µg/ml). The platinum concentration in peritoneal fluid at the start of HIPEC differed twofold between patients and was positively correlated with systemic exposure (*p* = .04) and peak plasma concentration (*p* = .04).

**Conclusion:**

In this exploratory study, no effect was found for postprocedural flushing on platinum concentrations in peritoneal tissue, blood, or drain fluid. BSA-based HIPEC procedure leads to large interpatient variability in platinum exposure in all compartments.

The study was registered at ClinicalTrials.gov on 7 December 2017 under registration number NCT03364907.

## Introduction

Peritoneal metastasis of colorectal origin is identified in 5–10% of patients undergoing primary resection, and metachronous colorectal peritoneal metastasis occurs in 20–50% of patients during follow-up [1–3]. Despite the use of modern systemic chemotherapy regimens, patients with peritoneal metastasis of colorectal cancer have poor outcome with a median overall survival of 10–16 months [4, 5]. Since the introduction of cytoreduction combined with hyperthermic intraperitoneal chemotherapy (HIPEC), median overall survival increased to 32–41 months [6–10].

The rationale for HIPEC is to obtain high local drug concentrations and high penetration in tumour tissue with relatively low systemic exposure. The response of tumour cells is dependent on drug concentration in peritoneal fluid. In organoids derived from colorectal peritoneal metastases, a platinum concentration of 118–275 µg/ml in peritoneal fluid is required to eliminate 50% of tumour cells during 30-min HIPEC procedure [11]. Although these findings cannot be easily extrapolated to in vivo tumour nodules in patients, it provides insight in the importance of the drug concentration in peritoneal fluid. As diffusion is the most dominant mechanism to penetrate in tissue for low-molecular-weight drugs, such as cisplatin and mitomycin C, higher drug concentration in peritoneal fluid results in higher drug concentration in tumour tissue [12]. Unfortunately, the optimal tissue concentration that is required to eliminate peritoneal metastases is unknown. It seems reasonable to strive for the highest local tissue concentration while limiting systemic exposure to prevent toxicity to the patient and the treating personnel in the postoperative period.[13, 14].

Although cytoreductive surgery (CRS) procedures are more or less standardised, large variations exist in HIPEC treatment modalities [15]. Important methodological variations include: technique (open ‘coliseum’ vs. closed abdomen), temperature, type and dose of the drug, exposure time, type and volume of carrier solution, and whether or not the peritoneum is flushed with crystalloids at the end of HIPEC. It is pivotal to understand the effects of these different variations on pharmacokinetics and pharmacodynamics of the treatment. Postprocedural flushing is predominantly performed with the idea to minimise platinum concentration in blood and decrease platinum concentration in drain fluid after surgery, resulting in lower personnel exposure risk. On the other hand, it might decrease peritoneal tissue concentration and as such decrease efficacy of the treatment. If there is an effect of postprocedural flushing on platinum concentrations in tissue, blood, or drain fluid, this may affect efficacy and safety of the treatment.

The primary goal of this exploratory study was to evaluate the effect of flushing with NaCl 0.9% on platinum concentration in peritoneal tissue, blood, and drain fluid after oxaliplatin-based HIPEC. In addition, the interindividual variability in tissue, blood, and drain fluid was explored and the relation between platinum concentration in peritoneal fluid at the start of HIPEC and platinum exposure in tissue and blood was investigated.

## Materials and methods

### Patients

Patients ≥ 18 years old with a diagnosis of preoperatively identified primary or recurrent peritoneal metastasis of colorectal origin who were planned for HIPEC treatment with oxaliplatin according to routine clinical care were eligible for study entry. Patients were sequentially allocated over both groups, meaning that ten patients were enrolled in the flushing group, followed by ten patients in the non-flushing group.

The GUTOX trial was approved by the institutional ethics committee Arnhem-Nijmegen (Nijmegen) and was compliant with the Declaration of Helsinki. All patients provided written informed consent before entering the study. The study was registered at ClinicalTrials.gov, NCT03364907.

### Study design

**Fig. 1:**
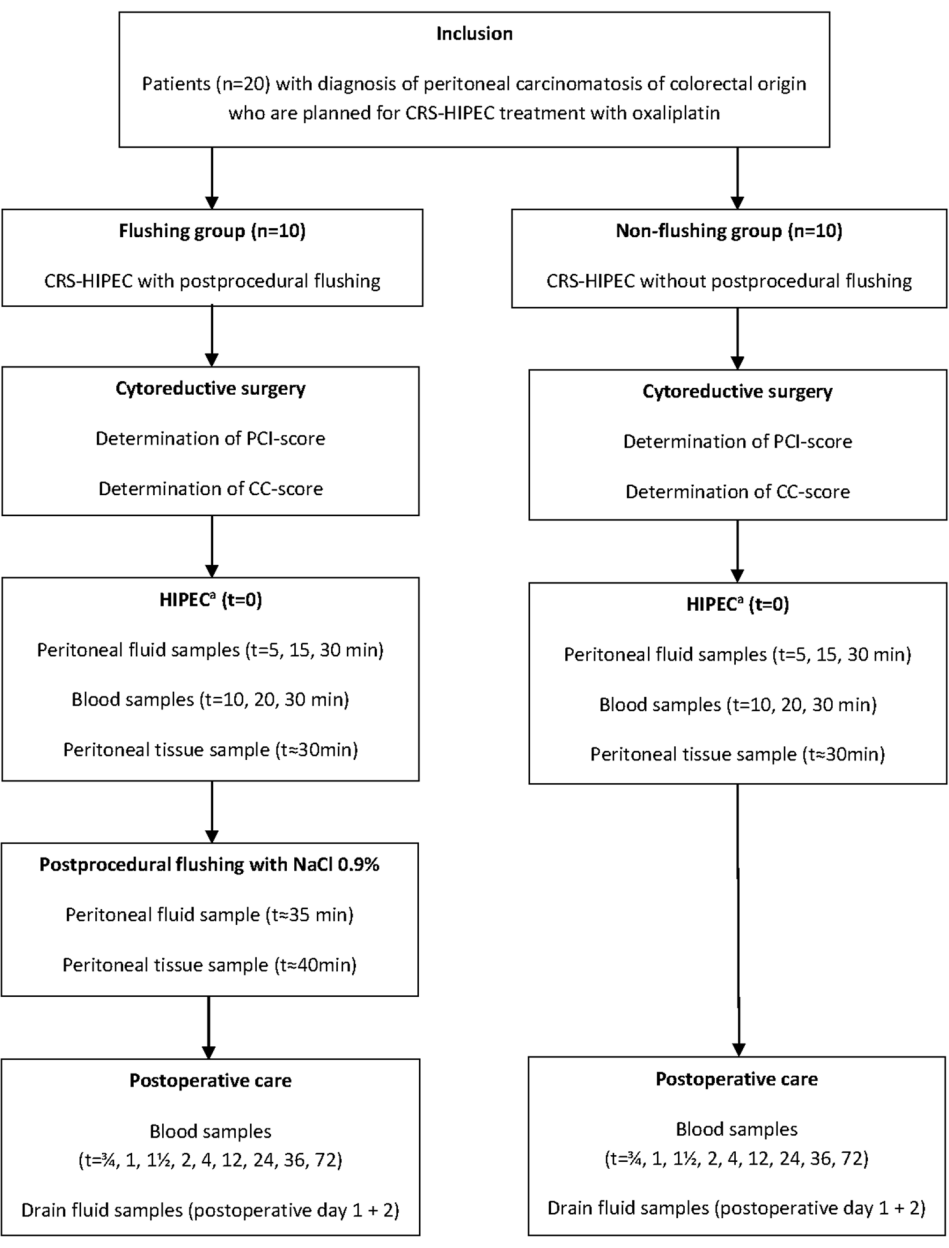
Study design. *CRS* cytoreductive surgery, *HIPEC* hyperthermic intraperitoneal chemotherapy, *PCI score* Peritoneal Cancer Index score, *CC score* completeness of cytoreduction score. ^a^HIPEC was performed with oxaliplatin 460 mg/m^2^ at 42–43 °C for a duration of 30 min

The GUTOX study was an exploratory, single-center, prospective, pharmacokinetic cohort study. The study design is graphically displayed in Fig. 1. In the flushing group, ten patients were treated with HIPEC including flushing afterwards. Flushing consisted of rinsing the abdominal cavity with 0.9% (w/v) sodium chloride immediately after the intraperitoneal chemotherapy was drained out of the abdominal cavity. In the non-flushing group, ten patients underwent HIPEC without flushing afterwards. Surgical procedure was performed according to the local routine protocol for CRS-HIPEC procedure [16]. Oxaliplatin-based HIPEC was performed using the open coliseum technique at a dose of 460 mg/m^2^, at a target temperature of 42–43 ℃ for a total duration of 30 min. Dextrose 5% was used as carrier solution. The volume of dextrose 5% was dependent on the abdominal volume which differed between patients. A flow rate of 1.2–2 L/min was used to circulate the perfusate. At the end of the chemoperfusion, the instillation solution was drained from the abdominal cavity.

### Pharmacokinetic sampling and analytical assay

Pharmacokinetic sampling was performed as described in Fig. 1. At the end of HIPEC, a small peritoneal tissue sample (~ 1 × 2 cm) from the dorsal side of the posterior rectus sheath was collected in patients of both groups. In the patients treated in the flushing group, a second peritoneal sample was collected immediately after flushing with crystalloids. Peritoneal tissue pre-treatment was performed according to an earlier described method [14]. After HIPEC, four drainage tubes were fixed to drainage bags to collect outflowing drain fluid during the postoperative period as described in Fig. 1. The total volume of drain fluid per bag was noted, starting the morning after HIPEC. Immediately after sampling, the drain bags were changed. All samples were stored at − 40 ℃ until analysis. Platinum concentrations were measured using flameless atomic absorption spectrometry according to a previously described method [17].

For non-compartmental pharmacokinetic analysis, Phoenix WinNonlin® version 8.1 (Certara USA Inc, Princeton, NJ) was used.

### Haematologic toxicity

The occurrence of haematologic toxicity was monitored for up to 14 days or until hospital discharge. Haematological lab monitoring took place as part of routine clinical care. Leukopenia, anaemia, and thrombocytopenia were graded according to Common Terminology Criteria for Adverse Events (CTCAE) v5.0 [18].

### Statistics

Statistical analysis was performed using SPSS version 25.0 (IBM Corp., Armonk, NY, USA). Pharmacokinetic parameters were described as geometric mean with range. Unpaired *t* tests were used to test for differences in patient characteristics with the exception of sex and CC score where a Chi-square test was performed. A paired sample *t* test was used on logarithmic transformed data to compare platinum tissue concentrations before and after postprocedural flushing in the flushing group. Unpaired *t* tests were used on logarithmic transformed data to compare platinum tissue concentrations, systemic exposure, and platinum in drain fluid between both groups. A Fisher’s exact test was used to compare haematologic toxicity between both groups. Spearman’s rank correlation tests were used to test for correlations between platinum concentration in peritoneal fluid, tissue exposure, and unbound and total systemic exposure. *p* < 0.05 were considered as statistically significant.

## Results

### Patients

Twenty patients were included in the GUTOX trial between March 2018 and June 2019. Patient characteristics are summarised in Table 1. Despite a non-randomised study design, patient characteristics were equally distributed.

**Table 1:** Patients’ characteristics

	Flushing (*n* = 10)	Non-flushing (*n* = 10)	*p* value
Age (year)	65 ± 15	63 ± 11	0.815
Sex (M/F)	6/4	3/7	0.178
Body mass index (kg/m^2^)	26.6 ± 3.8	28.4 ± 4.8	0.356
Body surface area (m^2^)	1.90 (1.76–2.10)	1.97 (1.80–2.12)	0.590
PCI score	4 (2–13)	8 (5–13)	0.423
CC score			
CC-0	10	9	0.305
CC-1	0	1	
Karnofsky score	90 (90–90)	90 (90–90)	1.000
Peritoneal metastasis (primary/recurrent)	10/0	10/0	1.000
eGFR using CKD-EPI (ml/min/1.73m^2^)	87 ± 5	88 ± 4	0.559

Data are presented as mean ± SD or as median (IQR)

*PCI score* Peritoneal Cancer Index score, *CC score* completeness of cytoreduction score, *eGFR* estimated glomerular filtration rate, *CKD-EPI* Chronic Kidney Disease Epidemiology Collaboration

### Peritoneal tissue

**Fig. 2:**
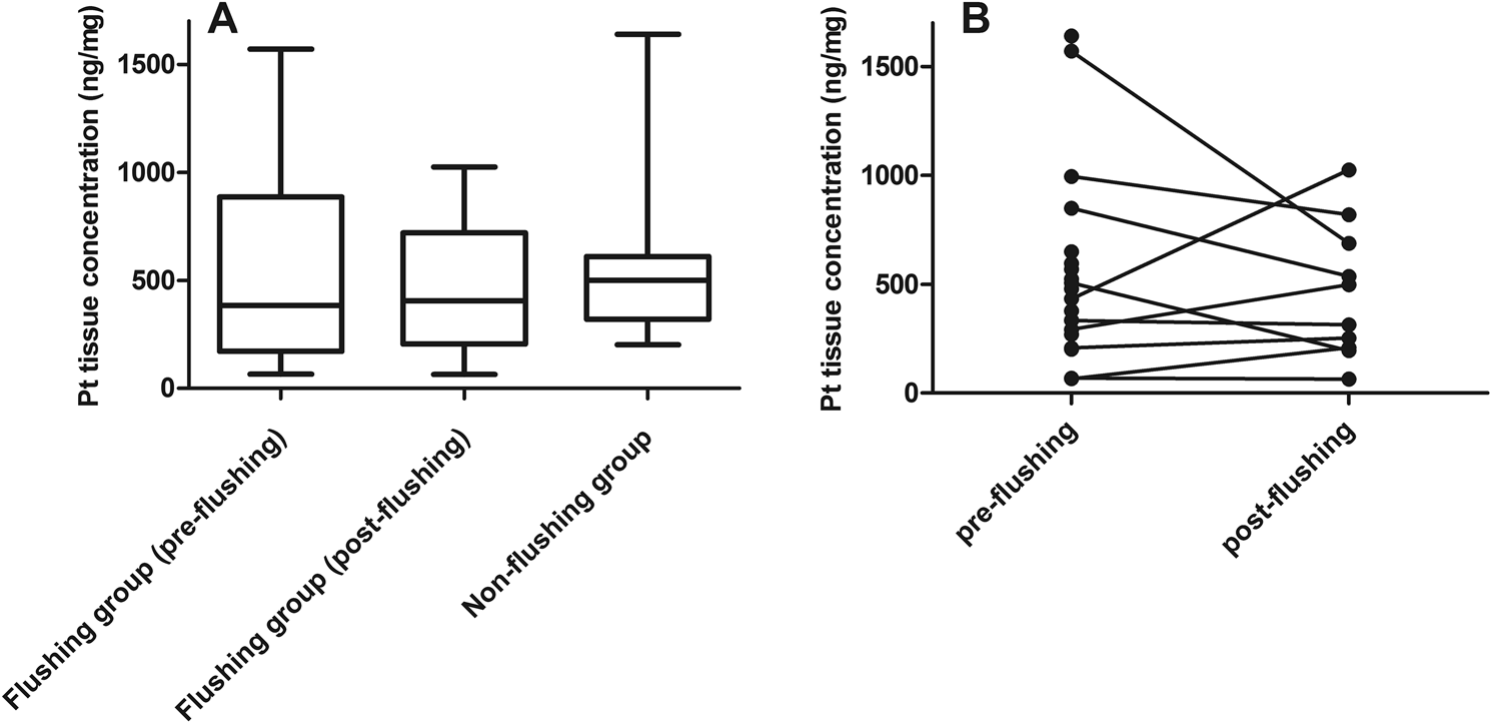
Platinum exposure in peritoneal tissue. **a** Boxplots of platinum peritoneal tissue concentration in patients of the flushing and the non-flushing group. **b** Individual platinum peritoneal tissue concentrations showing individual differences between pre- and post-flushing. Peritoneal tissue concentrations of patients of the non-flushing group are presented together with the pre-flushing tissue concentrations. *Pt* platinum

The interpatient variability in platinum concentrations in peritoneal tissue was substantial, ranging from 65–1640 µg/g dry weight. In the patients of the flushing group, no difference was found between the geometric mean [range] platinum concentrations in peritoneal tissue before and after postprocedural flushing (348 [66–1571] µg/g dry weight vs. 356 [65–1025] µg/g dry weight, respectively; *p* = 0.927). The platinum tissue concentrations pre- and post-flushing are graphically displayed in Fig. 2. The non-flushing group showed similar geometric mean [range] platinum concentrations in peritoneal tissue compared to the flushing group before postprocedural flushing (478 [202–1640] µg/g dry weight vs. 348 [66–1571] µg/g dry weight, respectively; *p* = 0.416).

### Blood

**Fig. 3:**
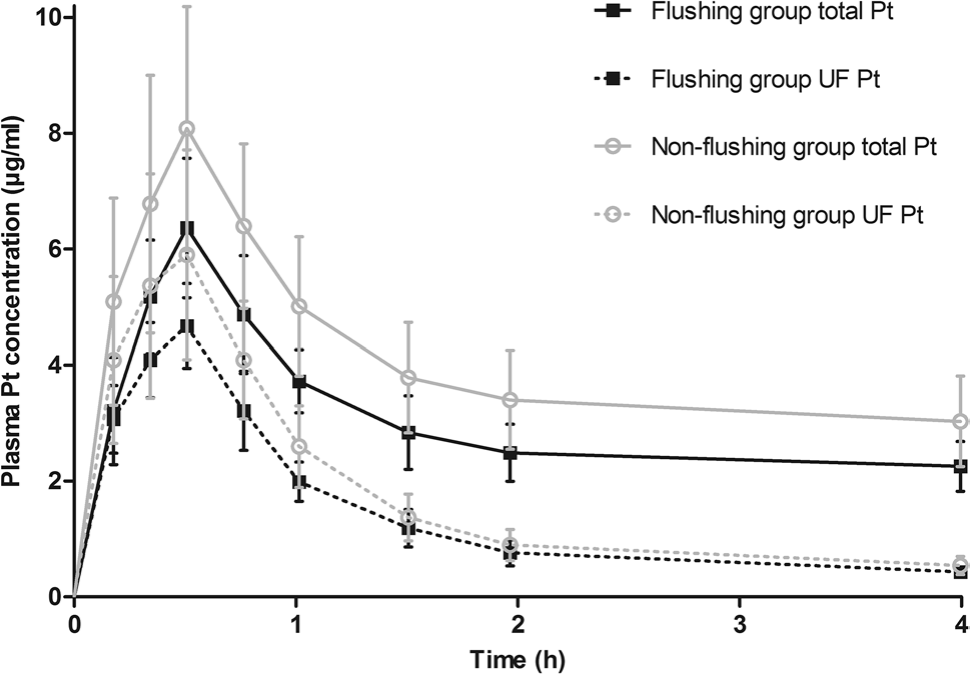
Plasma concentration–time curve during first 4 h including the 30 min of HIPEC. Data are presented as mean ± SD, *Pt* platinum, *UF Pt* ultrafiltered platinum

Interpatient variability in systemic exposure of ultrafiltered platinum and total platinum was 30% and 25%, respectively. Systemic exposure for ultrafiltered platinum and total platinum ranged from 10.5 to 28.0 µg*h/ml and 62.3 to 168.4 µg*h/ml, respectively. The patients of the flushing group showed lower geometric mean [range] C_max_ for both ultrafiltered platinum and total platinum than the patients of the non-flushing group (C_max_ total platinum: 6.3 [5.0–8.6] µg/ml vs. 8.0 [5.5–11.6] µg/ml, respectively; *p* = 0.024 and C_max_ ultrafiltered platinum: 4.6 [3.5–5.7] µg/ml vs. 5.9 [3.4–8.7] µg/ml, respectively; *p* = 0.043). Geometric mean [range] systemic exposure of total platinum was lower in the flushing group compared to the non-flushing group (90.4 [62.3–105.8] µg*h/ml vs. 116.4 [79.5–168.4] µg*h/ml, respectively; *p* = 0.019). There was no difference in geometric mean [range] systemic exposure of ultrafiltered platinum between both groups (14.8 [10.5–20.2] µg*h/ml vs. 18.1 [10.8–28.0] µg*h/ml, respectively; *p* = 0.141). Platinum pharmacokinetics of unbound and total platinum in plasma during HIPEC and the first 4 h post-HIPEC are shown in Fig. 3.

### Drain fluid

The geometric mean [range] of platinum cleared via drainage during the first 2 days after HIPEC did not differ between both groups (4.5 [2.6–7.7] mg vs. 6.3 [3.0–11.8] mg, respectively; *p* = 0.054). No differences were found between both groups in geometric mean [range] platinum concentration in drain fluid on day 1 post-HIPEC (7.6 [2.8–21.1] µg/ml vs. 7.7 [3.8–14.6] µg/ml, respectively; *p* = 0.953) or day 2 post-HIPEC (1.7 [0.7–5.4] µg/ml vs. 1.9 [1.4–3.1] µg/ml, respectively; *p* = 0.523). The platinum cleared via drainage appeared to be only a minor part (approximately 0.6%) of the totally administered dose.

### Peritoneal fluid

**Fig. 4:**
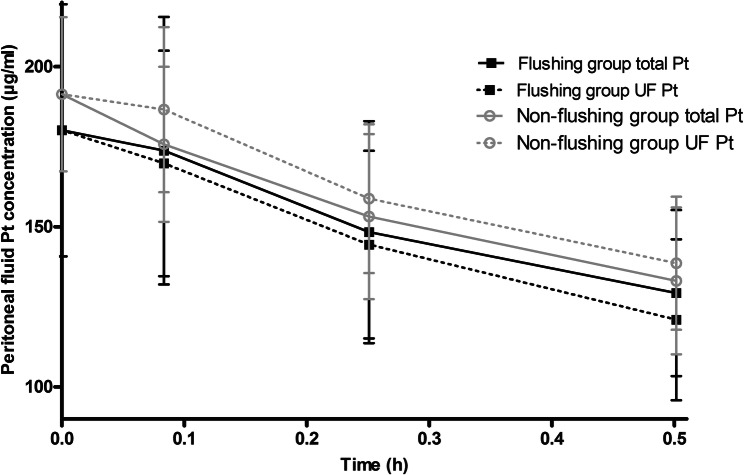
Peritoneal fluid concentration–time curve during HIPEC. The start concentration at timepoint 0 is a theoretical concentration calculated using the administered dose and the total peritoneal fluid administered. Data are presented as mean ± SD, *Pt* Platinum, *UF Pt* ultrafiltered platinum

Platinum concentration at start of HIPEC showed substantial interpatient variability, ranging from 122—246 µg/ml. On average, the total volume of peritoneal fluid, platinum concentration at start, and total exposure over 0–30 min in peritoneal fluid did not differ between the two groups. The peritoneal fluid concentration–time curve during HIPEC is shown in Fig. 4. All the platinum in peritoneal fluid was unbound platinum. During the 30-min oxaliplatin perfusion, total platinum concentration in peritoneal fluid decreased from 180 (± 39) µg/ml to 129 (± 26) µg/ml in the flushing group and from 191 (± 24) µg/ml to 133 (± 23) µg/ml in the non-flushing group, reflecting a decrease of approximately 28% and 30%, respectively. Postprocedural flushing decreased platinum concentration to negligible concentrations of 8 µg/ml, which is ~ 4% of the concentration at start.

### Correlations

Spearman’s correlation tests showed that ultrafiltered platinum concentration in peritoneal fluid at the start of HIPEC was positively correlated with both total systemic exposure (*p* = 0.04) and peak plasma concentration (*p* = 0.04), and negatively correlated with perfusate volume (*p* =  < 0.01). The peak ultrafiltered platinum plasma concentration was negatively correlated with perfusate volume (*p* = 0.04). No correlations were found between platinum concentration in peritoneal fluid at the start of HIPEC and tissue exposure.

**Fig. 5:**
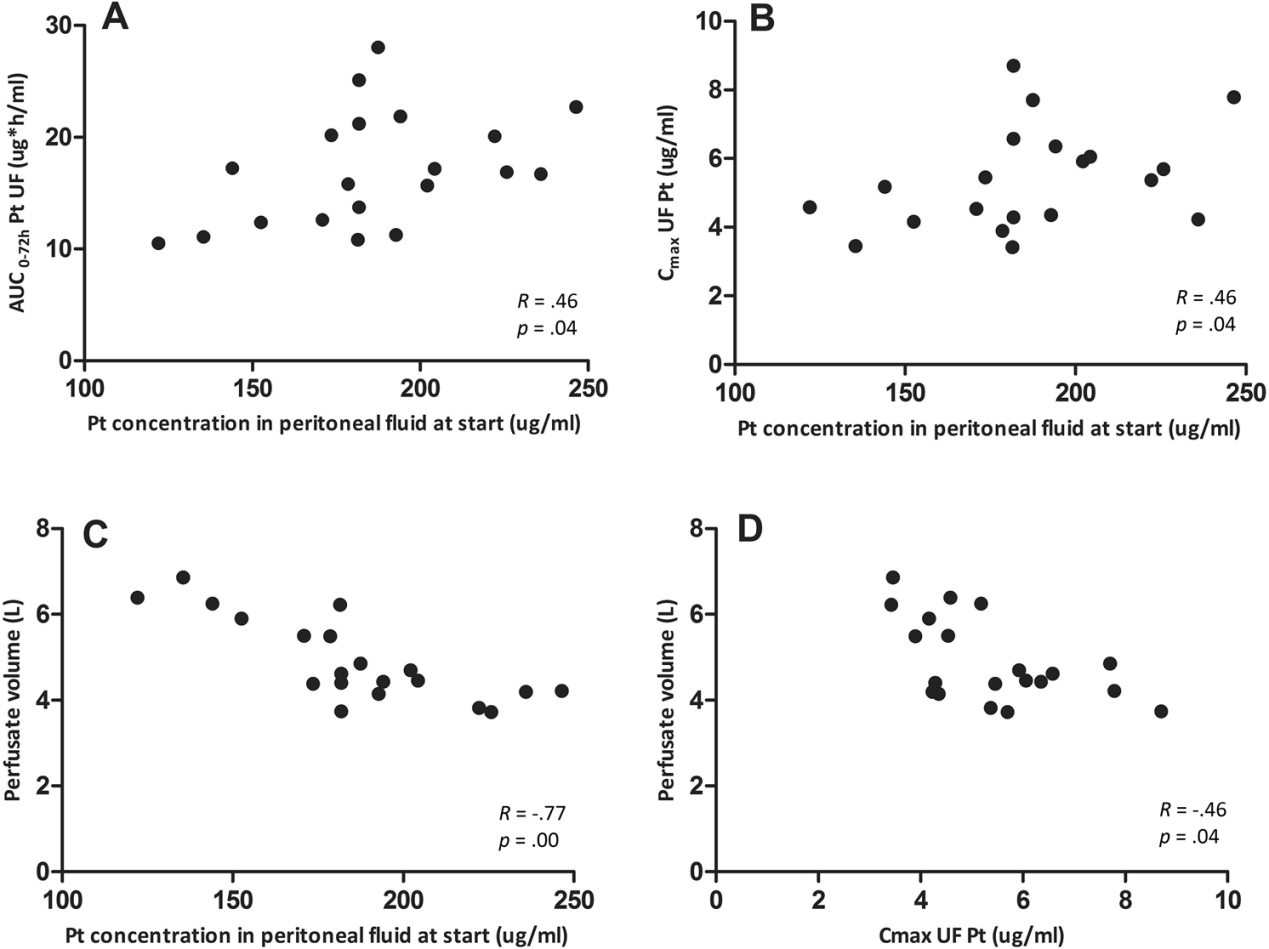
Statistical significant correlations. Scatterplots with Spearman’s correlation coefficient (*R*) and Sig(2-tailed) p value (*p*). Pt concentration in peritoneal fluid at start of HIPEC is positively correlated with both AUC_0-72 h_ Pt UF (**a**) and C_max_ UF Pt (**b**) in plasma and negatively correlated with perfusate volume (**c**). C_max_ UF Pt in plasma is negatively correlated with perfusate volume (**d**). *Pt* Platinum, *UF Pt* ultrafiltered platinum, *AUC*_*0-72 h*_ Area under the curve from 0 to 72 h, *C*_*max*_ peak plasma concentration

Pharmacologic parameters are summarised in Table 2. Correlations between the described variables are shown in a scatterplot in Fig. 5.

**Table 2:** Pharmacologic parameters

	Flushing (*n* = 10)	Non-flushing (*n* = 10)	*p* value
General information			
Oxaliplatin dose (mg)	877 ± 83	902 ± 124	0.603
Intravenous fluid administered between 1 h prior to HIPEC until the end of HIPEC (ml)	1193 ± 568	1114 ± 592	0.764
Peritoneal tissue			
Pt concentration in tissue pre-flushing (µg/g dry weight)	348 [66–1571]	478 [202–1640]	0.416
Pt concentration in tissue post-flushing (µg/g dry weight)	356 [65–1025]	–	0.927^a^
Plasma			
C_max_ total Pt (µg/ml)	6.3 [5.0–8.6]	8.0 [ 5.5–11.6]	0.024*
AUC_0-72 h_ total Pt (µg*h/ml)	90.4 [62.3–105.8]	116.4 [79.5–168.4]	0.019*
AUC_0-inf_ total Pt (µg*h/ml)	137.2 [77.2–209.2]	169.8 [117.6–285.2]	0.110
C_max_ UF Pt (µg/ml)	4.6 [3.5–5.7]	5.9 [3.4–8.7]	0.043*
AUC_0-72 h_ UF Pt (µg*h/ml)	14.8 [10.5–20.2]	18.1 [10.8–28.0]	0.141
AUC_0-inf_ UF Pt (µg*h/ml)	15.5 [11.1–21.9]	18.8 [11.2–29.0]	0.151
Drain fluid			
Volume produced during first 2 days (ml)	1142 [460–2280]	1312 [650–3025]	0.524
Pt cleared during first 2 days after HIPEC (mg)	4.5 [2.6–7.7]	6.3 [3.0–11.8]	0.054
Pt concentration on day 1 after HIPEC (µg/ml)	7.6 [2.8–21.1]	7.7 [3.8–14.6]	0.953
Pt concentration on day 2 after HIPEC (µg/ml)	1.7 [0.7–5.4]	1.9 [1.4–3.1]	0.523
Peritoneal fluid			
Volume of perfusate (ml)	5077 ± 1163	4755 ± 756	0.474
Pt concentration at the start of HIPEC (µg/ml)	180 ± 39	191 ± 24	0.450
Pt concentration at the end of HIPEC (µg/ml)	129 ± 26	133 ± 23	0.734
Pt concentration after flushing (µg/ml)	8 ± 8	–	–
AUC_0-0.5 h_ peritoneal fluid (µg*h/ml)	73.1 [48.6–109.6]	75.9 [63.7–100.4]	0.664

Data are presented as mean ± SD or as geometric mean [range]

*Pt* Platinum, *AUC*_*0-0,5 h*_ Area under the concentration–time curve from 0 to 0,5 h, *C*_*max*_ peak plasma concentration, *AUC*_*0-72 h*_ Area under the concentration–time curve from 0 to 72 h, *AUC*_*0-inf*_ Area under the concentration–time curve from 0 to infinite time, *UF Pt* ultrafiltered platinum

*P* values < 0.05 are considered statistically significant and are flagged with one asterisk (*)

^a^based on paired *t* test

### Haematologic toxicity

No difference was found in haematological toxicity between both groups (Table 3). None of the patients developed leukopenia. Any grade anaemia occurred in 90% of all patients (100% of the patients in the flushing group vs. 80% in the non-flushing group). Four patients in the flushing group and one patient in the non-flushing group experienced grade 3 anaemia. The median [range] time to nadir anaemia appeared 5 [1–10] days post-HIPEC in the flushing group and 3 [1–12] days post-HIPEC in the non-flushing group. Grade 1 thrombocytopenia occurred in 30% of the patients in both treatment groups. Median [range] time to nadir thrombocytes was 2 [1–4] days post-HIPEC.

**Table 3:** Haematologic toxicity

	Flushing (*n* = 10)	Non-flushing (*n* = 10)	*p* value
Leukopenia			
Any	0	0	1.000
Severe (grade 3 and 4)	0	0	1.000
Anaemia			
Any	10	8	0.474
Severe (grade 3 and 4)	4	1	0.303
Thrombocytopenia			
Any	3	3	1.000
Severe (grade 3 and 4)	0	0	1.000

## Discussion

Substantial interpatient variability was demonstrated for platinum concentrations in both peritoneal tissue, blood, and drain fluid during and after HIPEC procedure. Assuming that platinum exposure correlates with efficacy and safety of the treatment, the observed interpatient pharmacokinetic variability might affect treatment outcome and should be reduced. It is important to know which variations in HIPEC procedure contribute to high variability in platinum exposure. The GUTOX study evaluates the effect of postprocedural flushing on pharmacokinetics of oxaliplatin. In this exploratory study, no significant effect of flushing after HIPEC on platinum concentration in peritoneal tissue, blood, or drain fluid was detected, although a yet unexplained difference in systemic exposure between patients who were and were not flushed after the HIPEC procedure was observed.

Since platinum concentrations were prospectively collected in multiple compartments, including peritoneal fluid, peritoneal tissue, blood, and drain fluid, and this study provides insights in the further understanding of oxaliplatin distribution during HIPEC procedure. High platinum concentration in microscopic remnants of tumour after cytoreduction is important for efficacy of HIPEC [11, 19]. Platinum tissue concentrations in healthy peritoneal tissue instead of tumour tissue were measured, because tumour nodules need to be resected prior to HIPEC to give the patient probably most benefit from the procedure. This is a feasible approach, since Elias et al. showed in earlier studies that platinum tissue concentration after HIPEC is similar between tumour and healthy peritoneal tissue [14].

The analytical method used to quantify platinum in this study was not validated for measurement of platinum in peritoneal tissue and drain fluid. Nevertheless, it is unlikely that matrix will influence the analysis using the technique of atomic absorption spectrometry, since, with this technique, the total sample is burned at high temperature to vaporise and atomise elements including platinum that is being analysed. Patients were not randomised over both treatment groups, but were sequentially allocated to the flushing- and non-flushing groups. The local HIPEC protocol that was used for routine clinical care at the beginning of the study included postprocedural flushing. After the inclusion of the first ten patients in this study, the local HIPEC protocol was changed to HIPEC without postprocedural flushing. This indicates that all HIPEC patients, regardless of whether they participated in this study, were treated conform the prevailing local HIPEC protocol. Although no randomisation was performed, the patient characteristics were well balanced between both treatment groups. Additionally, no stratification was performed for factors that may influence platinum concentration in tissue, e.g., volume of perfusate and peritoneal fluid concentration, since these factors cannot be determined before the procedure.

Interpatient variability in platinum concentration in dried peritoneal tissue was high, which is in line with earlier findings [12]. The median tissue concentrations found in this study match with the results of Elias et al. who found a peritoneal platinum tissue exposure of 392 µg/g dry weight, using a similar HIPEC method [14]. HIPEC performed with a lower dose of 300 mg/m^2^ resulted in a notable lower peritoneal tissue concentration of only 50 µg/g dry weight [range 5–203 µg/g dry weight] [20]. In the GUTOX study, a correlation between platinum concentration in peritoneal fluid and peritoneal tissue could not be demonstrated. However, others demonstrated that higher platinum concentrations in peritoneal fluid resulted in higher tissue exposure [12]. Oxaliplatin was dosed based on BSA which resulted in a large range of platinum concentrations in peritoneal fluid of 122–246 µg/ml. From a pharmacological point of view, the use of a fixed drug concentration should be preferred when performing HIPEC. Concentration-based HIPEC should be incorporated in global standardisation of HIPEC protocols, which is unfortunately still not the case. Nevertheless, even when HIPEC is performed with a fixed oxaliplatin concentration, the interpatient variety is still high [12]. This suggests that in addition to peritoneal platinum concentrations, other factors will influence tissue exposure and thereby efficacy of the treatment.

The peak plasma concentration of total and ultrafiltered platinum was reached at the end of HIPEC and rapidly dropped after removing oxaliplatin from the abdominal cavity. The higher peak concentration and exposure over time for platinum in the non-flushing group was caused by a difference in absorption of platinum in the first 30 min of HIPEC, as can be seen in Fig. 3. The observed difference cannot be explained by an effect of flushing, because the procedure for both groups did not differ during the first 30 min of the HIPEC procedure. The observed difference could neither be explained by differences in renal function, platinum concentration in peritoneal fluid, absolute dose, extent of surgery, nor the amount of intravenous fluid administered around HIPEC procedure. Therefore, the observed difference is yet unexplained and needs further attention.

Interpatient variability in systemic exposure is considered moderate and is comparable with between-patient variability of 33% after intravenous administration [21], while others report lower interpatient variability for total platinum and ultrafiltered platinum of 12% and 4–15%, respectively [22]. The unbound platinum concentration is generally considered as the pharmacologically active moiety [21, 23]. The peak plasma concentration of ultrafiltered platinum observed in the GUTOX study (flushing: 4.6 and non-flushing: 5.9 µg/ml) after intraperitoneal administration of oxaliplatin in a dose of 460 mg/m2 was higher than the peak plasma concentration of ultrafiltered platinum observed after a 2-h intravenous infusion of oxaliplatin at a dose of 130 mg/m^2^ (1.21 µg/ml) [21]. This suggests a faster systemic absorption, which seems pharmacological plausible when administering a higher dose over a shorter time period. More important, the average total exposure over time for ultrafiltered platinum observed in the GUTOX study (15.5 and 18.8 µg*h/ml) is higher than the total systemic exposure for ultrafiltered platinum after a single 2-h infusion of oxaliplatin at 130 mg/m^2^ (11.9 µg*h/ml) [21]. Nevertheless, no oxaliplatin induced haematologic toxicity was identified in this study. Anaemia is a very common complication in the immediate postoperative period being present in up to 90% of patients after major surgery [24]. Usually, leukocytes and platelets reach their nadir within 7–14 days after chemotherapy. Erythrocytes live for approximately 120 days [25] and, therefore, will not reach a nadir for several weeks after treatment. In this study, nadir anaemia was observed after a median time of 3–5 days post-HIPEC. Therefore, the anaemia observed in this study should be contributed to the operative procedure and is unlikely related to oxaliplatin.

The most important contamination sources during postoperative care of the patients is the personnel exposure to urine and drain fluid from an HIPEC-patient. The concentration in drain fluid on day 2 was about five times lower compared to day 1. The platinum concentration in drain fluid on both days did not differ between the groups. However, a trend towards a difference in the absolute amount of platinum cleared via drain fluid was found. This can be explained by a higher total volume of produced drain fluid in the non- flushing group. The risk for personnel exposure is considered to be related with the platinum concentration and not with the absolute amount in drain fluid [26]. These results are in line with earlier findings reporting ranges in platinum concentrations in drain fluid of 0.6–13.2 µg/ml on day 1 post-HIPEC and 0.2–3.2 µg/ml on day 2 post-HIPEC [27]. These data underline the importance of safety precautions when handling drain fluid of HIPEC patients. Postprocedural flushing after HIPEC does not seem to reduce the risk for personnel exposure.

On average, no difference was found between total volume of perfusate and platinum concentration in peritoneal fluid at start of HIPEC between both groups. Although it is important to notice that the platinum concentration in peritoneal fluid differed up to twofold between individual patients (122 vs. 246 µg/ml), which might affect antitumour activity at the peritoneal level. The decline of free platinum in peritoneal fluid is mainly the result of absorption from the peritoneal compartment towards peritoneal tissue and the systemic compartment. Reactions with erythrocytes and other cell types or debris in perfusate are unlikely to occur. All platinum in peritoneal fluid consisted of free unbound platinum. A decrease of approximately 30% in total platinum concentration in peritoneal fluid was found which is consistent with the literature reporting decreases of 30–50% [12, 28].

In this exploratory study, no effect was found for postprocedural flushing after oxaliplatin-based HIPEC on platinum concentrations in peritoneal tissue, blood, or drain fluid. A detrimental effect of flushing on efficacy or safety of the treatment seems unlikely and, therefore, the use of postprocedural flushing should be debated to simplify the HIPEC procedure. This study showed that BSA-based HIPEC procedure leads to large interpatient variability in platinum exposure in all compartments. Assuming that exposure correlates with treatment outcome, the observed sources of variability in platinum exposure need to be further investigated.

## Data Availability

The data that support the findings of this study are available on request from the corresponding author. The data are not publicly available due to their containing information that could compromise the privacy of research participants.
