# Correction to: Wide variation in tissue, systemic, and drain fluid exposure after oxaliplatin‑based HIPEC: results of the GUTOX study

**DOI:** 10.1007/s00280-022-04450-2

**Published:** 2022-06-22

**Authors:** Loek A. W. de Jong, Fortuné M. K. Elekonawo, Marie Lambert, Jan Marie de Gooyer, Henk M. W. Verheul, David M. Burger, Johannes H. W. de Wilt, Etienne Chatelut, Rob ter Heine, Philip R. de Reuver, Andre J. A. Bremers, Nielka P. van Erp

**Affiliations:** 1 Department of Pharmacy, Radboud Institute for Health Sciences, Radboud University Medical Center (RUMC), P.O. Box 9101, 6500 HB Nijmegen, The Netherlands; 2 Department of Radiology and Nuclear Medicine, Radboud Institute for Health Sciences, Radboud University Medical Center (RUMC), P.O. Box 9101, 6500 HB Nijmegen, The Netherlands; 3 Department of Surgery, Radboud Institute for Health Sciences, Radboud University Medical Center (RUMC), P.O. Box 9101, 6500 HB Nijmegen, The Netherlands; 4 Institut Claudius‑Regaud, IUCT‑Oncopole, and CRCT, Université de Toulouse, Inserm, 1, avenue Irène Joliot‑Curie, Toulouse, France; 5 Department of Medical Oncology, Radboud Institute for Health Sciences, Radboud University Medical Center (RUMC), P.O. Box 9101, 6500 HB Nijmegen, The Netherlands

## Correction to: Cancer Chemotherapy and Pharmacology (2020) 86:141–150 10.1007/s00280-020-04107-y

This is an Erratum concerning a correction in the units for all results that were expressed as platinum concentration. The reported results were incorrectly labelled as total and free platinum concentrations, whereas they correspond to total and free oxaliplatin concentrations. This applies to the following sections of the article: abstract, material and methods, results, discussion, Figs. 2–5 and Table 2. Any reader who would convert these results of oxaliplatin concentrations to platinum concentrations should multiply the reported values by 0.49 (based on the molecular mass of both oxaliplatin and platinum which is 397.29 g/mol and 195.08 g/mol, respectively). Although the main conclusions of the article remain intact some parts of the discussion should be nuanced.

In the discussion (page 148) it is mentioned that the median tissue concentrations found in this study match with the results of Elias et al. who found a peritoneal platinum tissue concentration of 392 µg/g dry weight. In fact the median tissue concentrations in the GUTOX study is approximately half of the concentration reported in the study by Elias et al. However, taking into account the large interpatient variability the results remain in line with each other.

In the discussion (page 148), it is mentioned that the peak plasma concentration of ultrafiltered platinum observed in the GUTOX study after intraperitoneal administration of oxaliplatin in a dose of 460 mg/m^2^ was higher than the peak plasma concentration after a 2-h intravenous infusion of oxaliplatin at a dose of 130 mg/m^2^. This statement still accounts after conversion, although the difference in peak plasma concentration becomes smaller. The statement that average total exposure over time for ultrafiltered platinum observed in the GUTOX study (15.5 and 18.8 μg*h/ml) is higher than the total systemic exposure for ultrafiltered platinum after a single 2-h infusion of oxaliplatin at 130 mg/m2 (11.9 μg*h/ml) is incorrect after conversion since the actual ultrafiltered platinum exposure (7.6 and 9.2 μg*h/ml) is lower compared to 11.9 μg*h/ml. The lower systemic platinum exposure also explains the absence of haematological toxicity found in this study.

